# Supplementary material for: Changes of bacterial and fungal communities and relationship between keystone taxon and physicochemical factors during dairy manure ectopic fermentation
Source: PLoS One. 2022 Dec 19;17(12):e0276920. doi: 10.1371/journal.pone.0276920 (PMC9762577; doi:10.1371/journal.pone.0276920)

PLOS ONE

Changes of bacterial and fungal communities and relationship between keystone taxon and physicochemical factors during dairy manure ectopic fermentation

--Manuscript Draft--

**Manuscript Number:**	PONE-D-22-13572
**Article Type:**	Research Article
**Full Title:**	Changes of bacterial and fungal communities and relationship between keystone taxon and physicochemical factors during dairy manure ectopic fermentation
**Short Title:**	Bacterial and fungal communities change and the relationship with physicochemical factors during EFS
**Corresponding Author:**	Erguang Jin<br>Wuahan Academy of Agricultural Sciences<br>Wuhan, CHINA
**Keywords:**	
**Abstract:**	During manure ectopic fermentation, the composition and diversity microbiota is constant changing because of interactions with fluctuating environmental and physicochemical factors. This study investigated changes in bacteria and fungi in dairy manure ectopic fermentation, which was aimed to identify primary taxon and relationship between keystone species and physicochemical characteristics. Changes of bacterial and fungal diversity and compositions were studied by 16S rRNA and 18S rRNA gene sequencing, respectively. Our results showed dominant phyum were Chloroflexi, Firmicutes, Proteobacteria, Bacteroidetes, and Actinobacteria of bacteria, and Ascomycota of fungi during the composting with a difference in relative abundances. Community diversity of bacterial and fungal fluctuated significantly and inversely during fermentation. Correlation between physicochemical parameters and community keystone taxon were analyzed, which showed closely relationship, and C/N ratio was significantly negative related to keystone genus. These findings indicated that microbial composition and diversity were interacted with fermentation environment and parameters, and management of physicochemical factors and microorganism could contribute to maturation efficiency and quality promotion during dairy manure ectopic fermentation. Our research would be helpful to improve the quality and efficiency of dairy farm waste handling.
**Order of Authors:**	Ping Gong
	Xiuzhong Hu
	Junjun Tan
	Lijun Wu
	Wu Liu
	Yu Yang
	Erguang Jin
**Additional Information:**	
**Question**	**Response**
**Financial Disclosure**<br><br>Enter a financial disclosure statement that describes the sources of funding for the work included in this submission. Review the submission guidelines for detailed requirements. View published research articles from *PLOS ONE* for specific	The funders had no role in study design, data collection and analysis, decision to publish, or preparation of the manuscript.

Powered by Editorial Manager® and ProduXion Manager® from Aries Systems Corporation

examples.<br><br>This statement is required for submission and **will appear in the published article** if the submission is accepted. Please make sure it is accurate.<br><br>**Unfunded studies**<br>Enter: *The author(s) received no specific funding for this work.*<br><br>**Funded studies**<br>Enter a statement with the following details:<br>• Initials of the authors who received each award<br>• Grant numbers awarded to each author<br>• The full name of each funder<br>• URL of each funder website<br>• Did the sponsors or funders play any role in the study design, data collection and analysis, decision to publish, or preparation of the manuscript?<br>• **NO** - Include this sentence at the end of your statement: *The funders had no role in study design, data collection and analysis, decision to publish, or preparation of the manuscript.*<br>• **YES** - Specify the role(s) played.<br><br>* typeset	
**Competing Interests**<br><br>Use the instructions below to enter a competing interest statement for this submission. On behalf of all authors, disclose any competing interests that could be perceived to bias this work—acknowledging all financial support and any other relevant financial or non-financial competing interests.<br><br>This statement is required for submission and **will appear in the published article** if the submission is accepted. Please make sure it is accurate and that any funding sources listed in your Funding Information later in the submission form are also declared in your Financial Disclosure statement.	The authors have declared that no competing interests exist.

Powered by Editorial Manager® and ProduXion Manager® from Aries Systems Corporation

View published research articles from *PLOS ONE* for specific examples.<br><br>**NO authors have competing interests**<br><br>Enter: *The authors have declared that no competing interests exist.*<br><br>**Authors with competing interests**<br><br>Enter competing interest details beginning with this statement:<br><br>*I have read the journal's policy and the authors of this manuscript have the following competing interests: [insert competing interests here]*<br><br>* typeset	
**Ethics Statement**<br><br>Enter an ethics statement for this submission. This statement is required if the study involved:<br><br>• Human participants<br>• Human specimens or tissue<br>• Vertebrate animals or cephalopods<br>• Vertebrate embryos or tissues<br>• Field research<br><br>Write "N/A" if the submission does not require an ethics statement.<br><br>General guidance is provided below. Consult the submission guidelines for detailed instructions. **Make sure that all information entered here is included in the Methods section of the manuscript.**	N/A

**Format for specific study types**<br><br>**Human Subject Research (involving human participants and/or tissue)**<br>• Give the name of the institutional review board or ethics committee that approved the study<br>• Include the approval number and/or a statement indicating approval of this research<br>• Indicate the form of consent obtained (written/oral) or the reason that consent was not obtained (e.g. the data were analyzed anonymously)<br><br>**Animal Research (involving vertebrate animals, embryos or tissues)**<br>• Provide the name of the Institutional Animal Care and Use Committee (IACUC) or other relevant ethics board that reviewed the study protocol, and indicate whether they approved this research or granted a formal waiver of ethical approval<br>• Include an approval number if one was obtained<br>• If the study involved *non-human primates*, add *additional details* about animal welfare and steps taken to ameliorate suffering<br>• If anesthesia, euthanasia, or any kind of animal sacrifice is part of the study, include briefly which substances and/or methods were applied<br><br>**Field Research**<br><br>Include the following details if this study involves the collection of plant, animal, or other materials from a natural setting:<br>• Field permit number<br>• Name of the institution or relevant body that granted permission	
**Data Availability**<br><br>Authors are required to make all data underlying the findings described fully available, without restriction, and from the time of publication. PLOS allows rare exceptions to address legal and ethical concerns. See the PLOS Data Policy and FAQ for detailed information.	Yes - all data are fully available without restriction

Powered by Editorial Manager® and ProduXion Manager® from Aries Systems Corporation

A Data Availability Statement describing where the data can be found is required at submission. Your answers to this question constitute the Data Availability Statement and **will be published in the article**, if accepted.<br><br>**Important:** Stating 'data available on request from the author' is not sufficient. If your data are only available upon request, select 'No' for the first question and explain your exceptional situation in the text box.<br><br>Do the authors confirm that all data underlying the findings described in their manuscript are fully available without restriction?	
**Describe where the data may be found in full sentences. If you are copying our sample text, replace any instances of XXX with the appropriate details.**<br><br>• If the data are **held or will be held in a public repository**, include URLs, accession numbers or DOIs. If this information will only be available after acceptance, indicate this by ticking the box below. For example: *All XXX files are available from the XXX database (accession number(s) XXX, XXX.).*<br>• If the data are all contained **within the manuscript and/or Supporting Information files**, enter the following: *All relevant data are within the manuscript and its Supporting Information files.*<br>• If neither of these applies but you are able to provide **details of access elsewhere**, with or without limitations, please do so. For example:<br><br>*Data cannot be shared publicly because of [XXX]. Data are available from the XXX Institutional Data Access / Ethics Committee (contact via XXX) for researchers who meet the criteria for access to confidential data.*<br><br>*The data underlying the results presented in the study are available from (include the name of the third party*	Data can be shared for reasonable request to corresponding author.

and contact information or URL).<br>• This text is appropriate if the data are owned by a third party and authors do not have permission to share the data.<br><br>* typeset	
Additional data availability information:	Tick here if the URLs/accession numbers/DOIs will be available only after acceptance of the manuscript for publication so that we can ensure their inclusion before publication.

Powered by Editorial Manager® and ProduXion Manager® from Aries Systems Corporation

Manuscript Click here to access/download;Manuscript;Plos one manuscripts 20220509.docx

Changes of bacterial and fungal communities and relationship between keystone taxon and physicochemical factors during dairy manure ectopic fermentation

Ping Gong, Xiuzhong Hu, Junjun Tan, Lijun Wu, Wu Liu,Yu Yang*, Erguang Jin*

Institute of Animal Husbandry and Veterinary, Wuhan Academy of Agricultural Science, 430208, Wuhan, Hubei, P.R.China

*Corresponding author: Yu Yang, E-mail: yang007yu@outlook.com; Erguang Jin, E-mail: jeg0617@126.com;

Abstract

During manure ectopic fermentation, the composition and diversity microbiota is constant changing because of interactions with fluctuating environmental and physicochemical factors. This study investigated changes in bacteria and fungi in dairy manure ectopic fermentation, which was aimed to identify primary taxon and relationship between keystone species and physicochemical characteristics. Changes of bacterial and fungal diversity and compositions were studied by 16S rRNA and 18S rRNA gene sequencing, respectively. Our results showed dominant phyum were *Chloroflexi*, *Firmicutes*, *Proteobacteria*, *Bacteroidetes*, and *Actinobacteria* of bacteria, and *Ascomycota* of fungi during the composting with a difference in relative abundances. Community diversity of bacterial and fungal fluctuated significantly and inversely during fermentation. Correlation between physicochemical parameters and community keystone taxon were analyzed, which showed closely relationship, and C/N ratio was significantly negative related to keystone genus. These findings indicated that

microbial composition and diversity were interacted with fermentation environment and parameters, and management of physicochemical factors and microorganism could contribute to maturation efficiency and quality promotion during dairy manure ectopic fermentation. Our research would be helpful to improve the quality and efficiency of dairy farm waste handling.

Keywords: Ectopic fermentation system, dairy manure, bacteria, fungi, network analysis

1. Introduction

During the last decades, the livestock industry has been developed fastly in China, meanwhile, the cattle stock achieved about 0.09 billion in 2018 [1]. With the rapid development of the dairy industry, the resultant cattle manure, especially large amount of dairy manure, was produced approximate 4×10^8 ton annually according to statistical data from China's Ministry of Agriculture. Untreated and arbitrarily dis-charged waste causes serious pollution in the surrounding environment, and also seriously restricts the sustainable and healthy development of the dairy industry [2, 3]. Moreover, intemperate emission of farm sewage can cause groundwater contamination challenges, which would also a huge threaten to environment and human health [3]. Hence, sustainable and efficient methods to deal with livestock manure pollution would be needed to handle the waste and the urgent concerns caused by livestock waste.

Ectopic fermentation systems (EFSs) have been used in dairy manure control, which involves the "dead bed" problem of in situ deep litter bedding, which could achieve harmless treatment and resource utilization of cow manure [3, 4]. Biological fermentation bed with chaff, pieces of straw, sawdust, mushroom residue, rice chaff, a moderate amount of microorganism strains as raw material, mixing after laying in the inner packing bed, cow excretion of feces to be absorbed by the padding and through microbial rapid decomposition, so as to achieve the aim of no manure emissions, after use, packing can be as raw materials for the production of organic fertilizer or soil use [2, 4, 5]. EFS was a novel method to treat both feces and urine efficiently, and environmentally friendly, and the discharged waste and post-fermentation padding

could be further used as bio-fertilizers [6].

In the process of manure fermentation, it is necessary to monitor whether the content of the main pathogenic microorganisms can meet the national organic fertilizer standards. Microbial fermentation bed has the effect of degrading manure, and the microorganism plays the role of material energy transformation, which is the core of the benign operation of the fermentation bed [7, 8]. As fermentation bed contains a rich microbial population, changes of community and function could affect the process and results [3, 9, 10]. On the other side, EFS could decrease the major manure based zoonotic bacteria, including *Salmonella spp*, *Campylobacter spp*, *Listeria monocytogenes*, *Yersinia enterocolitica*, *Escherichia coli* and *protozoa* [11]. Yang et al (2018) found that thermophilic bacteria especially Bacillus have an important role in EFS by 16S rRNA sequencing. Previous research indicated bacteria and fungi interaction with physiochemical factors could affect composting technology. However, litter research focus on bacteria and fungi interaction nor a long time dynamic change during EFS.

This study aimed to investigate the dynamic change of the main pathogenic microorganism, physicochemical property, microbiota and fungi during EFS. Our study was aim to study the bacterial and fungal diversity and composition and explore the influence of environmental and physicochemical parameters on the variation of bacteria and fungi during EFS of dairy manure with rice husk and rice chaff.

2. Materials and Methods

2.1 Experimental Materials

EFSs experiment was carried out in an ectopic fermentation bed with a total volume 1000 m^3 (48 m×12 m×1.8 m). Mixed materials were rice husk and rice chaff by volume ratio 6:4 as cow mattress bed. The main raw material was dairy mature and sewage mixture for ectopic fermentation. The experimental period was from 2019 Jun. 23 to Sep. 27. Ectopic fermentation samples at day 0, 1, 3, 8, 15, 22, 30, 37, 44, 51, 58, 65, 72, 86 and 93 were collected. On each sampling day, six samples were collected randomly from different sites of the fermentation substrate and immediately pooled. Then, the samples were divided into two parts on ice, and transferred to the laboratory. One part was stored at −80°C for microbial analysis by sequencing. The other part was used for the determination of physical and chemical properties of the EFS.

2.2 Detection of total Bacteria, Coliform bacteria, Salmonella, Staphylococcus aureus, and fecal Coliform in fecal contamination

Sewage samples were collected according to GB/T 25169-2010. The total number of mold, *Coliform*, *salmonella* and *Staphylococcus aureus* were detected by dilution plate counting method [12] with aseptic operation in ultra clean machine. 1 ml sewage (or 1 g waste) was draw into 9 ml saline and then sufficient oscillated. Pre-diluting mixed liquid to different gradient (10^{-2}, 10^{-3}, 10^{-4}, 10^{-5}, 10^{-6}, 10^{-7}), each 1 ml dilution from two or three adjacent samples was transferred to a petri dish with nutrient AGAR medium at 46 ℃. Each gradient had two repeats. After 72 h cultivating at 30 ℃, total *Bacteria* were counted. Two or three samples with adjacent dilutions were coated on the EMB plate, SS plate, and B-P plate, respectively, with 100 μL each. Each gradient

was repeated for two times. The samples were cultured at 37℃ for 24h. The number of *Coliform bacteria*, *Salmonella and Staphylococcu* were counted. Results were expressed as log value [lg (CFU/ mL)] of microbial quantity in 1g fecal contamination. The value of *fecal coliform* was determined by multiple-tube fermentation [13].

2.3 Analysis of physicochemical parameters

Fresh samples were used for measurement of moisture content, pH, NH_4^+-N (NN), total organic carbon (TO), total nitrogen (TN) and the C/N ratio using previously described methods in Chinese National Agricultural Organic Fertilizer Standard (NY525-2012) [14].

2.4 Microbial analysis

2.4.1 DNA Extraction and high- throughput sequencing

Microbial DNA was extracted from intestinal contents using a QIAamp DNA Stool Mini Kit (Qiagen, Germany) following the manufacturer's protocols. Successful DNA extraction was confirmed by 0.8% agarose-gel electrophoresis. Bacterial 16S rRNA and fungal 18S rRNA gene was amplified using primers 341F (5'-ACT CCT ACG GGA GGC AGC AG-3') and 806R (5'-GGA CTACHV GGG TWT CTA AT-3') and 547F (5'-CCAGCASCYGCGGTAATTCC-3') and V4R (5'-ACTTTCGTTCTTGATYRA-3') respectively. The PCR conditions were predenaturation at 98 ℃ for 2 min, 25 cycles of denaturation at 98 ℃ for 15 s, annealing at 55 ℃ for 30 s, elongation at 72 ℃ for 30 s, and a final post-elongation cycle at 72 ℃ for 5 min. The PCR products were purified with AMPure XP beads (AXYGEN). After

purification, the PCR products were used for the construction of libraries and then paired-end sequenced on Illumina Miseq (Illumina, CA, USA) at the Personalbio, Shanghai, China.

2.4.2 Sequence Filtering, OTU Clustering, and Sequence Analyses

The double-ended FASTQ sequences were filtered using the sliding window method, and then FLASH (v1.2.7, http://ccb.jhu.edu/software/FLASH/) was used to align the sequences that passed the quality filtering step [15]. The FASTA and QUAL files from Mothur were converted to FASTQ format using USEARCH (Version 7.0) [16]. Downstream processing and operational taxonomic unit (OTU) identification were performed using UPARSE [17]. Barcodes and primers from the merged sequences were removed. After dereplication and abundance sorting were performed, where singletons were retained, sequences with a minimum similarity of 97% were clustered into OTUs using the average neighbour algorithm. Alpha-diversity analyses, including community richness index (Chao1), community diversity index (Shannon), and community evenness (Pielou_e) determinations were performed using Mothur [18].

2.4.3 Network analysis

Microbiota sequencing data from EFS samples to perform network analysis. Absolute abundance data were used for correlation analysis in R to determine correlation between every genus and every other genus within each individual microbiota sample. To avoid the bias introduced by different microbial OTU numbers, we selected the relative abundances of the 100 most abundant genus for microbial and fungal group. Robust correlations between two genera were defined as those with

SparCC correlation coefficients > 0.85 and false discovery rate-corrected P-values < 0.01 for bacterial community and correlation coefficients > 0.6 and false discovery rate-corrected P-values < 0.05 for fungal community. The topology parameters including degree, centrality, clustering coefficient and average shortest path length of each network were determined in Cytoscape 3.8.0 using Network Analyzer [19].

2.5 Statistical analysis

Data on pathogenic microorganisms, physiochemical properties, alpha diversity were assessed by ANOVA. The procedure for the repeated measurements of SAS (SAS Institute, Inc., Cary, NC, United States) was used. Data were given as means ± standard errors of the means. A mean comparison was performed using the Duncan's Multiple Range test method with a significant level of $P < 0.05$. We used a nonparametric Mann–Whitney test to determine the variance of topology parameters between microbial and fungal network. In the figures, $P < 0.05$ indicates statistical significance (*$P < 0.05$, **$P < 0.01$, ***$P < 0.001$). Correlations were analyzed by using SparCC and correlation coefficients >0.5 and false discovery rate-corrected *P*-values < 0.05 were defined robust correlations between two variates. Analyses were performed using R (R Core Team, Vienna, Austria), Graphpad Prism (version 8.0.1, Graphpad Software Inc, La Jolla, California, USA) and SAS (version 9.4; SAS Inst. Inc., Cary, NC).

3. Results

3.1 Changes of main pathogenic microorganisms during the ectopic fermentation of cow manure

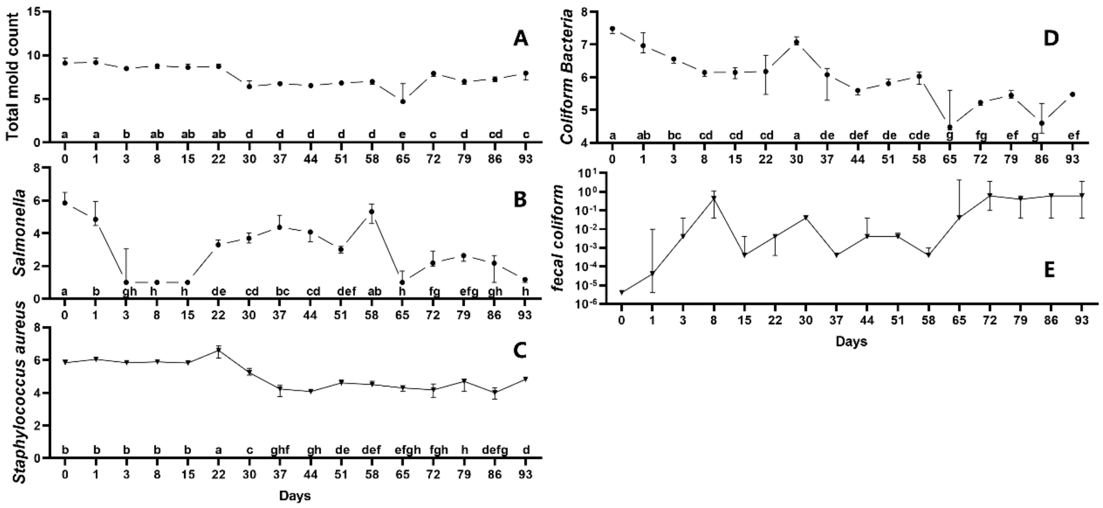

Figure 1. Changes of pathogenic bacteria and fecal coliform in ectopic fermentation bed. Count of total mold (A), *Salmonella* (B), *Staphylococcus aureus* (C), *Coliform Bacteria* (D) and *fecal coliform* (E) were assessed during the EFS process. Statistical assessment was carried out with one-way ANOVA followed by post hoc test using Dunn's multiple comparison test. Values with different letters are significantly different at $P < 0.05$.

The dynamic changes of count of total mold (A), Salmonella (B), Staphylococcus aureus (C), Coliform Bacteria (D) and fecal coliform (E) during ectopic fermentation bed process were shown in Figure 1. The count of total mold (Fig. 1A) at the early stage (0 d- 22 d) of the ectopic fermentation was significantly higher than that of other time period ($P < 0.01$). And, from day 30 to day 65 the count of total mold was reduced but then increased during day 72 d to day 93. During the fermentation process, the number of Salmonella fluctuated greatly, which decreased significantly from day 0 to day 8, and increased again from day 8 to day 37, after which decreased significantly at 65 days (Fig. 1B). It was eventually decrease in last 20 days and lower than initial day (Fig. 1A). The content of Staphylococcus aureus decreased during the fermentation process, reached its peak at 22d, and stabilized from 37d to 93 d (Fig. 1C). Although the number

of Coliform Bacteria of at day 30 was significantly higher than that of other periods ($P < 0.01$), it showed the gradually decrease during the fermentation process (Fig. 1D). Fecal coliform is the minimum sample size for detecting a fecal coliform unit, and is an important hygienic index for systematically evaluating the harmless effect of manure treatment [20]. In the ectopic fermentation bed process, the fecal coliform value appeared a peak at 8d, then decreased again, and increased significantly from 65d to 93d, and reached the highest value at 65d, 72d and 93d (Fig.1E). Thus, after ectopic fermentation, the risk of fecal coliform in cow manure could be reduced.

3.2 Analysis of changes in physicochemical characteristics during fermentation

Physicochemical characteristics of pH, TO, TN, C/N, moisture and NN were measured to indicate the fermentation process (Fig.2). The TO showed a fluctuation during the fermentation time, that a low level at first 3 days and stayed at a relative high level during day 8 to day 65, then dropping at last month (Fig.2B). Besides, TN gradually raised in the whole fermentation time, while C/N showed a successively decrease (Fig.2C and Fig.2D). In the first day of fermentation, the pH was initially decreased, and gradually increased to day 15, followed by another decrease to day 51. As the fermentation continued, pH showed slightly decrease from day 58 to day 79, followed by an increase from day 79 to 93 (Fig.2E). The NN was about 50 g/L at initial time, and there were two drop time at day 1 and day 37 respectively. During day 1 to day 30, the NN increased slowly and dropped from day 44 to day 93 (Fig.2F). Moisture of the bed gradually increased in first 22 days followed a fluctuation during day 22 to day 58, after which it slightly increased and eventually moisture was 71.14%(Fig.2G).

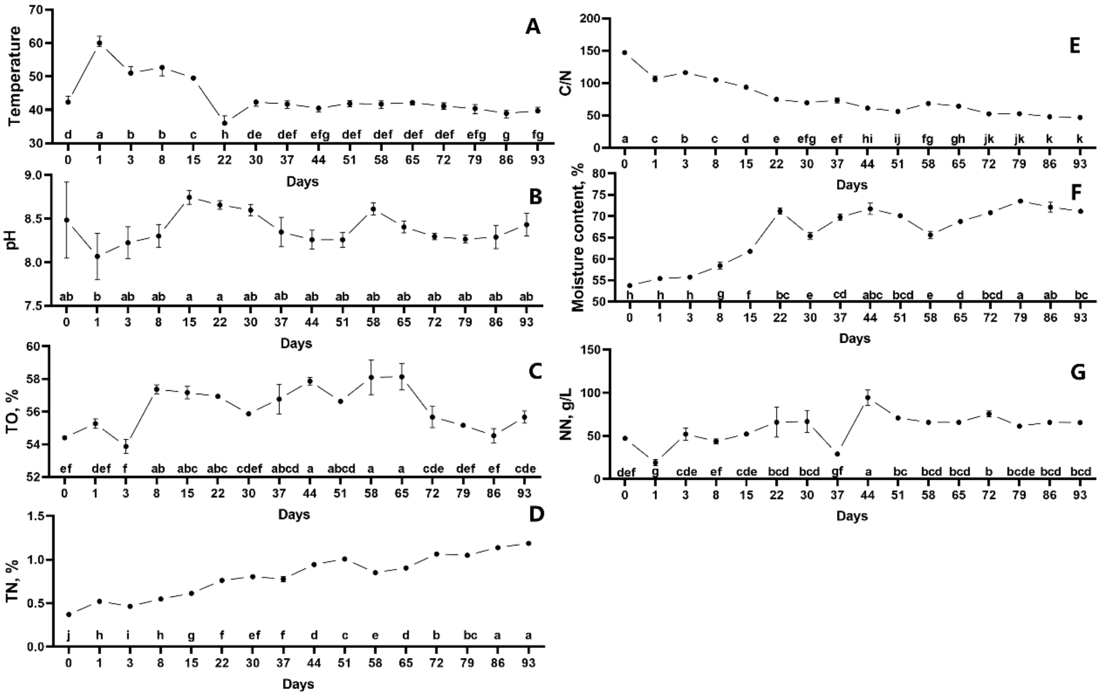

Figure 2 Changes in temperature (A), pH (B), TO (C), TN (D), C/N (E), moisture content (F) and NN (G) were assessed during the EFS process. Statistical assessment was carried out with one-way ANOVA followed by post hoc test using Dunn's multiple comparison test. Values with different letters are significantly different at $P < 0.05$.

3.3 Analysis of bacterial diversity in ectopic fermentation of cow manure

In order to illuminate the overall change of microbiota community, we measured the diversity changes during the EFS. Chao1 index showed a stable time during the early 8 days, and slightly increased from day 15 to day 86, with a little drop at day 93. Meanwhile, Shannon index showed a dramatic drop from day 0 to day 15, followed by an increase from day 15 to day 86 which also showed twice decline at day 58 and day 72 respectively. The Pielou_e index which indicates community evenness also showed an obvious descent at first 15days which increased from day 15 to day 22 followed by a plateau from day 22 to day 51, and increased from day 58 to day 86, although a drop

happened at day 72. On the other hand, β diversity was analyzed in PCoA based on Bray-Curtis distance which showed dramatic change from day 0 to day 22, especially from day 3 to day 15. The changes of β diversity from day 22 to day 93 were inconspicuous.

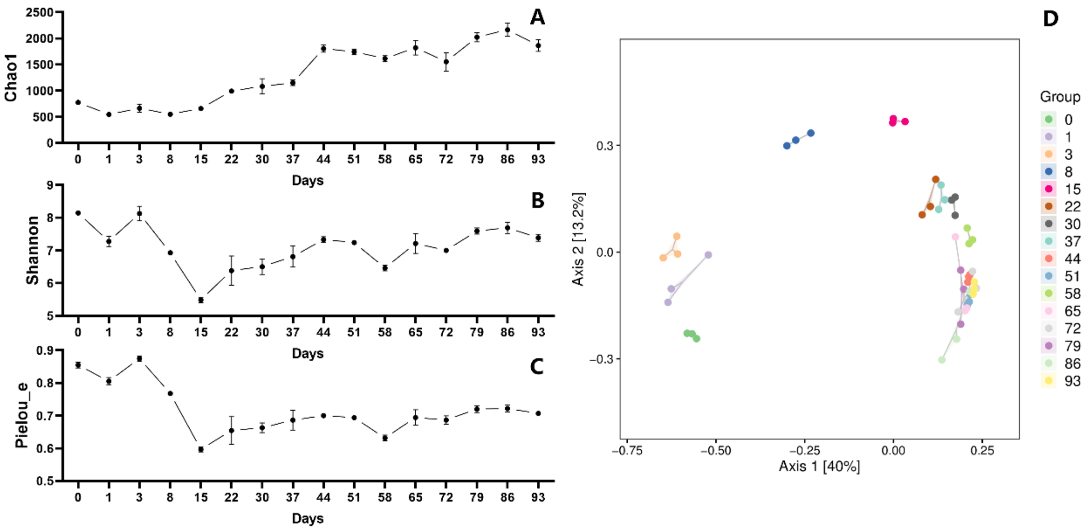

Figure 3 Changes in bacterial alpha and beta diversity during fermentation. Chao1 (A), Shannon (B), Pielou_e (C), and Principal coordinate analysis (PCoA) (E) were assessed during the EFS process. Statistical assessment was carried out with one-way ANOVA followed by post hoc test using Dunn's multiple comparison test. Values with different letters are significantly different at $P < 0.05$.

3.4 Analysis of bacterial community in ectopic fermentation of cow manure

Top five bacterial phyla were *Chloroflexi*, *Firmicutes*, *Proteobacteria*, *Bacteroidetes*, and *Actinobacteria*, which were accounted for over 90% of bacterium (Fig.4A). At beginning of EFS, *Firmicutes*, *Proteobacteria* and *Bacteroidetes* were

dominant in microbiota community, while they decreased and *Chloroflexi* significantly increased and turned dominant after EFS (Fig.4A). Meanwhile *Actinobacteria* was slightly promoted after EFS (Fig.4A). Specifically, the relative abundance of *Chloroflexi* reached its peak at 15 day of ectopic fermentation, and then gradually decreased (Fig.4A). Relative abundance of *Firmicutes* reached its peak at day 8, and then decreased gradually (Fig.4A). The relative abundance of *Proteobacteria* decreased gradually (Fig.4A). The relative abundance of *Bacteroidetes* decreased gradually, reached its lowest at 15 day of fermentation, and then gradually increased (Fig.4A). As shown in Fig.4B and Fig.4C, *Streptococcus*, *Prevotella*, *Lactobacillus*, *Bifidobacterium*, *Acinetobacter* and *Comamonas* was dominant at day 0, and *Clostridium*, *Ureibacillus*, *Coprococcus*, *Pseudoxanthomonas* and *Thermovum* was enriched in day 1 to day 8 (Fig.4B and Fig.4C). The *Sobibacillus* was enriched in day 22, while B-42, *Hydrogenophaga* and *Methylocaldum* was enriched in day 65 to day 79, and *vadinCA02*, *Thauera*, *Ruminofilibacter*, *Sedimentibacter* and *Syntrophomonas* was enriched in day 65 to 93 (Fig.4B and Fig.4C). Besides, dominant genus was *Clostridium*, *Thauera*, *Hydrogenophaga*, *Sedimentibacter* and *B-42* at the end of fermentation (Fig.4B and Fig.4C).

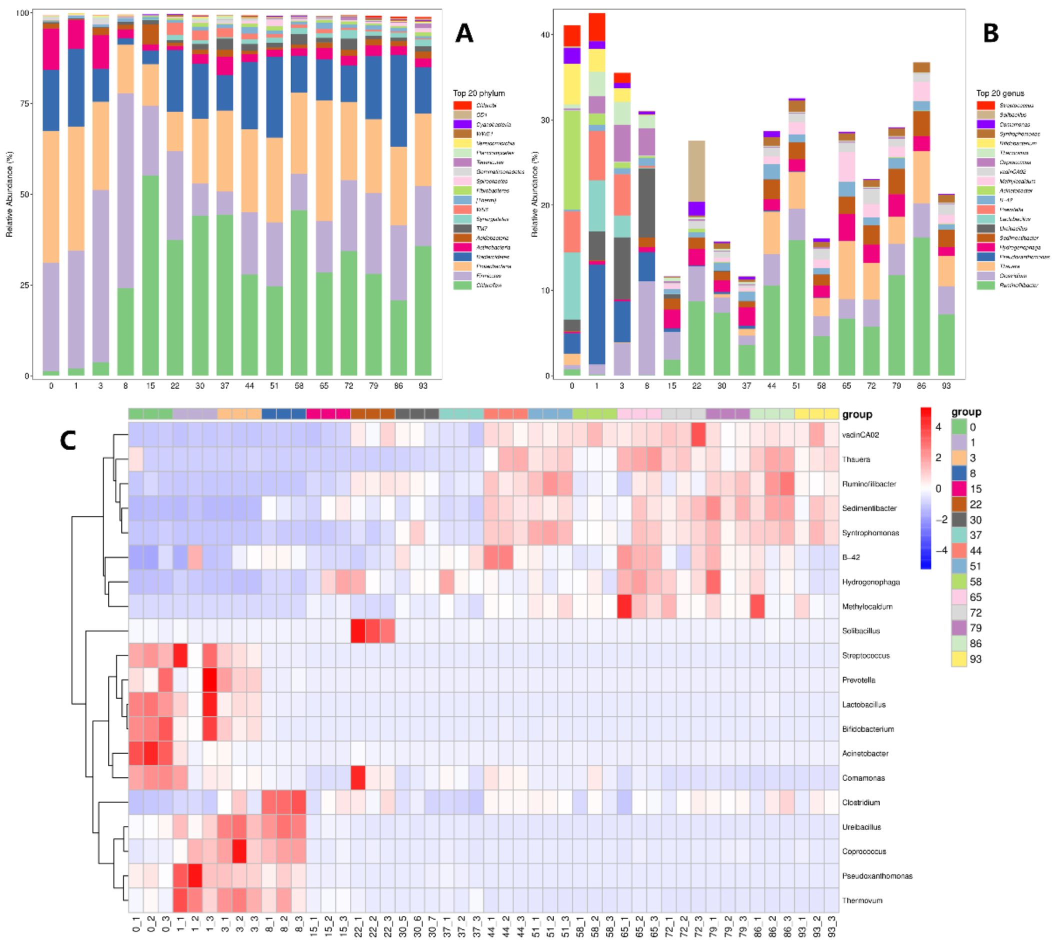

Figure 4 Changes in bacterial phylum and genus level and heatmap of top 20 genus in sample in ectopic fermentation of cow manure

3.5 Analysis of fungal diversity in ectopic fermentation of cow manure

The evolution of fungal diversity change was estimated by Chao1, Shannon and Pielou_e index in this research (Fig.5). Chao1 index maintained at a plateau from day 0 to day 8, followed by a dramatic drop from day 8 to day 22, and became to be nearly stable (Fig.5A). Meanwhile, Shannon and Pielou_e index showed a same trend like Chao1 index, which stayed indifference at first 8 days followed an obvious decreased in a week, and turned to a little change later (Fig.5B and Fig.5C). The β diversity was

measured by Principal Component Analysis (PCoA), which showed drastic change from day 3 to day 22, while the first 3 days and later time showed similar respectively.

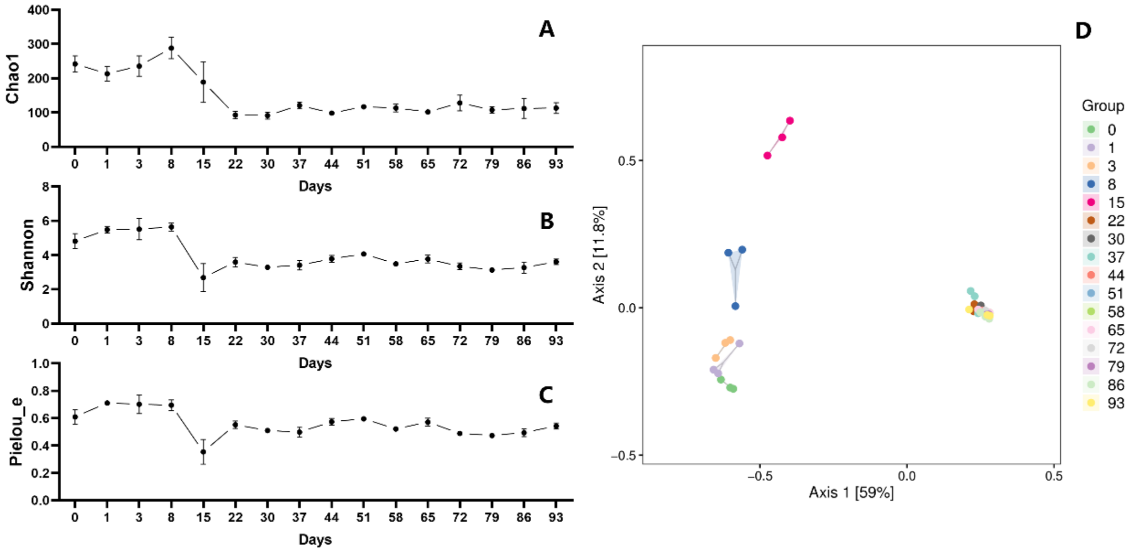

Figure 5 Changes in fungal alpha and beta diversity during fermentation. Chao1 (A), Shannon (B), Pielou_e (C), and Principal coordinate analysis (PCoA) (E) were assessed during the EFS process. Statistical assessment was carried out with one-way ANOVA followed by post hoc test using Dunn's multiple comparison test. Values with different letters are significantly different at $P < 0.05$.

3.6 Analysis of fungal community in ectopic fermentation of cow manure

At phylum level, the dominant fungi phyla were *Ascomycetes*, *Mucoromycota* and *Basidiomycota* at day 0 accounted more than 70% together, while *Mucoromycota* decreased from 30% to less than 5% from day 0 to day 8 and *Ascomycetes* showed slightly change. The *Basidiomycota* was increased in first 8days and also decreased from 3.22% at day 8 to nearly disappeared after day 22. The *Chytridiomycota* was increased from day 22 to day 51 and then disappeared. At genus level, *Aspergillus*, *Rhizormucor* and *Thermomyces* were dominant at day 0, and gradually decreased to

day15. Meanwhile, *Mycothermus* turned to increase rapidly reaching its highest level at day 15, while dramaticly dropped in day 22, also maintaining the dominant in the identified genus. The heatmap also showed changes of the main genus during EFS which mainly happened from day 0 to day 8. At day 0, *Aspergillus*, *Rhizomucor*, *Thermomyces*, *Tilletia*, *Moeszipmyces* and *Ustulagininoidea* was more enriched than other genus, and in day 1 *Issachenkia*, *Cephalotrichum* and *Fusarium* were slightly increased. From day 3 to day 8, the abundant genus turned to *Issachenkia*, *Pseudallescheria*, *Sarocladium*, *Lecanicillium* and *Simplicillium*. At day 8, only *Mycothermus* was the dominant genus consistant with fig 4. After day 8, all the genus showed a low abundance in the EFS, while most of the reads were unidentified.

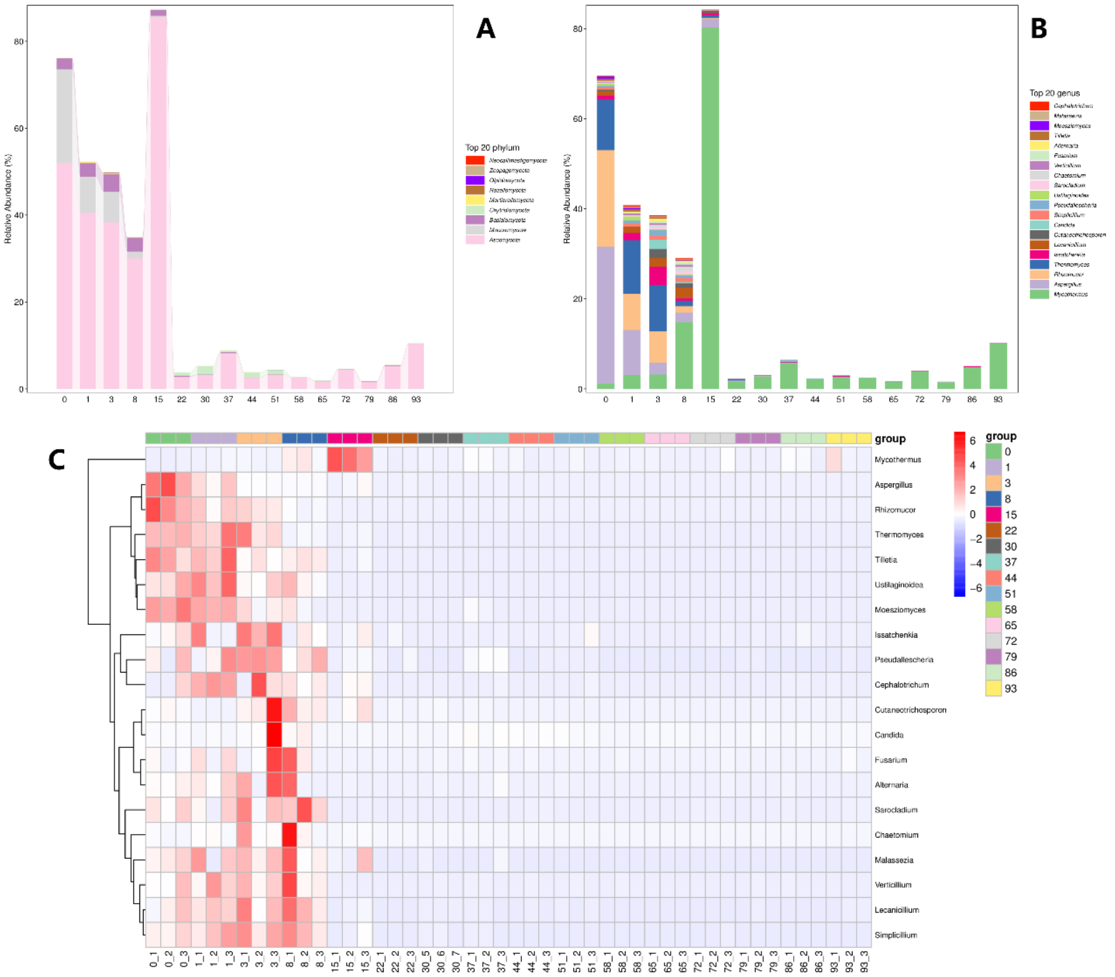

Figure 6 Changes in fungal phylum and genus level and heatmap of top 20 genus in sample in ectopic fermentation of cow manure

3.7 Network analysis of microbial communities and correlation between keystone genus and environmental factors

The co-abundance network was analyzed by SparCC for bacteria and fungi respectively. The bacteria showed a more complex network than fungi (Fig 7A-B), with 25 nodes and 57 edges in bacteria network and 10 nodes and 15 edges for fungi. The bacterial network showed higher degree, a more complicated network during fermentation, lower closeness centrality which indicates how close a node is to all other nodes in the network and higher average shortest path length. Based on the networks, we calculated key stones genus by MCC arithmetic in cytoHubba plugin in Cytoscape. *unidentified_Methylophilaceae*, *Pseudoxanthomonas*, *Thermovum*, *unidentified_Sphingobacteriaceae*, *unidentified_Bacillales*, *Ureibacillus*, *Streptococcus*, *Megasphaera*, *unidentified_Weeksellaceae*, and *Syntrophomonas* were the key stone genus in bacteria network, while the 10 fungal genus turned to be keystone genus in fungal network. Correlation analysis among keystone bacteria, fungi and environment factors showed moisture, C/N and TO were most relevant to bacteria and fungi, while *unidentified_Methylophilaceae*, *Ureibacillus* were most relevant to fungi and environment factors, and *Rhizormucor*, *Thermomyces* and *Aspergillus* were most relevant to bacteria and enrironment factors. These results indicated that keystone

bacteria and fungi were affected by each other and moisture, C/N and TO were the key environmental factors on bacteria and fungi changes during EFS.

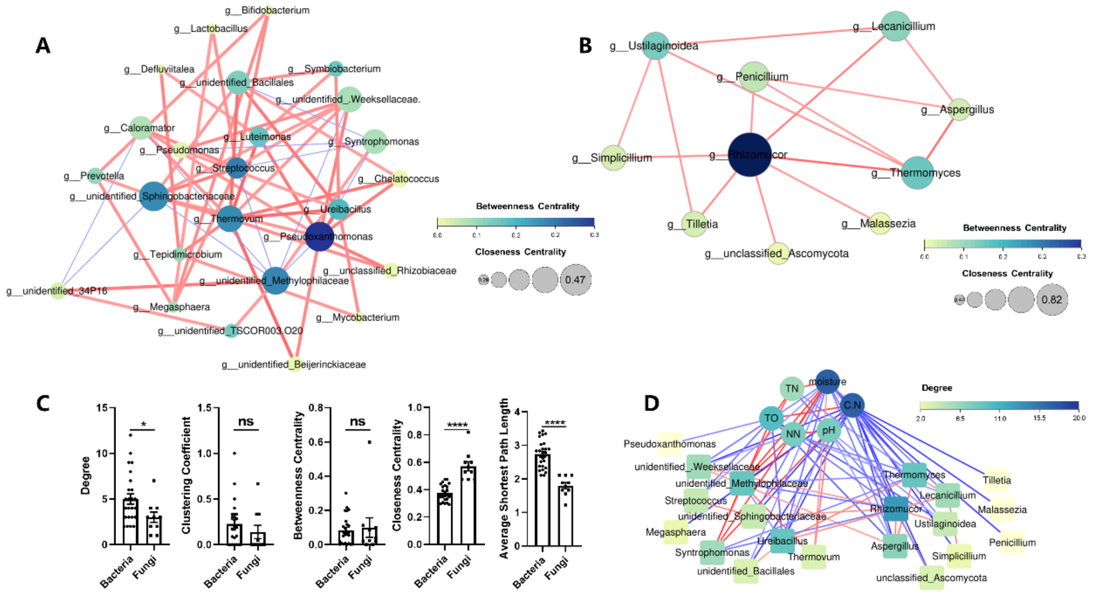

Figure 7 Network analysis of bacterial and fungal community and correlation between keystone genus and environmental factors. In bacterial (A) and fungal (B) network, nodes in the network represent taxon (genus level), and node size is proportional to closeness centrality, while node color is proportional to betweenness centrality; blue edge indicates negative correlation and red edge indicates positive correlation. Topological properties of nodes in bacterial and fungal networks (C). Correlation among keystone genus in bacterial and fungal network and environmental factors (D), and blue edge indicates negative correlation and red edge indicates positive correlation, while node color is proportional to degree.

3.8 Relationship between the bacterial and fungal community and environmental factors

Redundancy analysis was conducted to illustrate the correlation between the bacteria (Fig 8A) and fungi (Fig 8B) and environmental parameters (temperature, pH, TO, TN, C/N, moisture content and NN). Arrows in figures mean different environmental factors, and longer length of the rays are used to indicate extent of impact. The first axis explained 41.61% and 55.68% variation of diversity, and 11.9% and 5.1% of the variation were explained at the second axis, respectively. The seven studied environmental parameters were covered in the RDA biplot which means they all significantly influenced the EFS process. In the figures, angles between the environment factors and sample dots less than 90 represents a positive correlation. Additionally, smaller angles indicated stronger correlations. T and C/N had significant effect on bacterial and fungal community change during earlier 8 days, while moisture content, NN and TN had significant effect on day 44 to day 93.

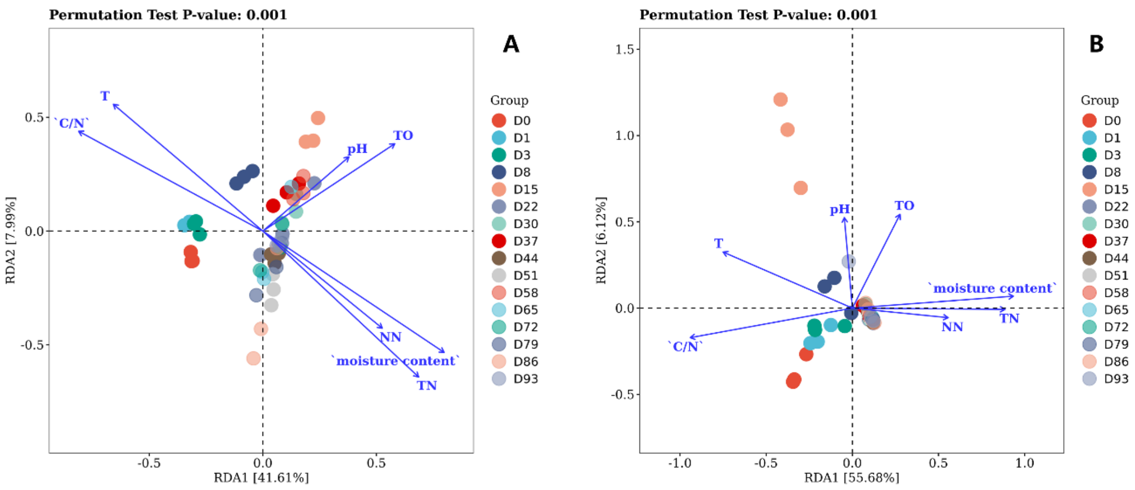

Figure 8 Redundancy analysis (RDA) used to assess the relationships of bacterial communities (A) and environmental factors, as well as fungal communities (B) and

environmental factors during the EFS process. The values of axes are the percentages explained by the corresponding axis.

4 Discussion

In this research, we showed the changes of bacterial and fungal community and their relationships with the physicochemical parameters which closely associated with these changes during dairy manure ectopic fermentation. The physicochemical factors in our work not only determined rates of many biological processes, but also play selective roles in the evolution and succession of microbiological communities [11]. Nitrogen and organic carbon are essential for microbial growth and energy production, and the change of C/N ratio would effect on microorganism growth [21]. Besides, the C/N ratio is considered an excellent indicator of the decomposition and fermentation, while decrease means a higher level of decomposition and fermentation maturity [10]. Thus, the results showed our fermentation achieved a higher level of decomposition and fermentation maturity. Urea in the ectopic fermentation system could be converted to NN, which would increase pH degree of the fermentation mixtures, on the other side, acids produced by microorganism led the decrease of pH through the composting [22]. Besides the moisture change due to evaporation of water would also influence the pH degree [21]. Our results showed pH change was consistent with NN to some degree, which means denitrification by microorganism would more important for pH change. By the way, changes of pH would also effect on the growth of microorganism and fermentation process [23]. Overall, the measurements showed our fermentation process

was matured and at a high level of decomposition based on the changes of physicochemical characteristics during fermentation. In addition, the results indicated microorganism bioactive would be important for the fermentation.

Alpha diversity index (Chao1, Shannon and Pielou_e index) describes the bacterial species richness, diversity and evenness, and reflects dynamic changes of microbial community during nitrogen transformation and surrounding changes during dairy manure ectopic fermentation [24]. The main trend of Chao1 index showed increased, which was opposite to C/N ratio. These results demonstrated that increased species richness would help the maturity of fermentation. The declining of community diversity and evenness of denitrifying bacteria in the system during fermentation would lead to more nitrogen retention under the form of nitrate and avoid excessive nitrogen release in the forms of NO, N_2O, and N2. In these gases, NO is toxic and N_2O is a major greenhouse gas, with nearly 300 times greater global warming potential than equivalent mass of CO_2 [25]. Although our results showed the overall community diversity changes, it also indicated the effect of reducing environment pollution. Besides, we found the main pathogenic microorganisms showed an overall downward trend with slightly fluctuating, and the fecal coliform was increased significantly after fermentation. Thus, after ectopic fermentation, the risk of pathogenic microorganisms and fecal coliform to environment from cow manure would be reduced.

With the changes of environmental factors and physicochemical characteristics during the fermentation, the initial dominant microbial taxa associated with the compost

materials were gradually changed. In our study, *Firmicutes* and *Proteobacteria* were the most dominant phylum at first 8 days, while *Firmicutes* was slightly increased. Previous study showed that *Firmicutes* was dominant and increased at early stage, which could adapt to different composting systems utilizing various organic wastes [26]. *Proteobacteria* was dominant at not only early stage, but also during the whole fermentation. It is said that many class or order of *Proteobacteria* could be enriched in various stage of fermentation, which indicated that some mesophilic bacteria became dormant at the thermophilic period and returned to growth at the cooling phase [27]. Also, *Proteobacteria* has been reported to degrade high-molecular-weight polysaccharides such as starch and cellulose. The *Chloroflexi* increased gradually and became the most dominant from day 15, and *Chloroflexi* contributed major function genes of nitrate reductase play key role in denitrification processes of reduction NO_3^--N to NO_2^--N and was positively correlated with NN [21].

Regarding to fungi, the most dominant phylum was Ascomycetes overall the composting process, while *Mycothermus*, *Aspergillus*, *Rhizormucor*, *Thermomyces* were enriched at first 3 days and *Mycothermus* become the most dominant fungal genus. *Thermomyces* was related to hemicellulose degradation [28, 29]. *Aspergillus* was the most abundant fungi in single-stage inoculation all over the process, indicating that it is important to degrade lignocellulose [28, 30]. Morevoer, *Aspergillus* was also main fungi during second high-temperature composting process based on herbal residue composting, and *Aspergillus* secreted cellulases and hemicellulases to decompose the residual lignocellulose following the thermophilic stage [31]. Besides, previous studies

also indicated that *Aspergillus* and *Mycothermus* were widely exist in composting fermentation [30, 32-34]. Besides, *Mycothermus* secreted hydrolases to participate in the degradation of cellulose and hemicellulose and was dominated during the thermophilic stage of cow manure composting [35]. In adition, *Mycothermus* was also dominant during thermophilic stage of swine manure and rice straw co-composting [36] and a thermophilic fungus commonly found in composting which could secret heat-resistant enzymes to degrade lignocellulose [37]. Taken together, fungi community played dominant and vital role in degrading various organic materials, and pointed out potential species for improving composting process effectively.

According to network analysis, bacterial network showed a higher average degree, which meant a more complex network and more coupled among all genus. Closeness centrality represents a node that is closed to all other nodes in the network and indicates the information transmission between nodes in a high speed and efficient [38], and the shortest path indicates how quickly information can travel between two nodes [39, 40] Thus fungi can respond to environmental changes more quickly than bacteria. Positive correlations were dominated in both the bacterial and fungal networks, which implied mutual cooperation played more important roles in microbial assembly during EFS; and There were more negative correlations in the bacterial network than that in the fungal network, which indicated frequent inter-competition in bacterial communities during EFS [41]. The co-occurrence network showed the glimpse of community interactions and showed that bacteria were more closeness than fungi during dairy manure ectopic fermentation. This suggested that bacterial communities kept a more stable status based

on corporation and competition, and disclosed the reason of lower fungi community diversity. The keystone bacterial and fungal genus was closely related to physicochemical factors indicated that changes of microorganism during fermentation drove the composting process.

Also, there are still some limitation of this study. Firstly, we did not set a control or treated to investigate effects of environment factors or physiochemical properties on the composting process. Secondly, the analysis of bacteria and fungi were based on 16S rRNA and 18S rRNA gene sequence, which were adequate to disclose accurate species, strains, and microbial functions. We did not culture the potential key species and verified their function about improving parameters for monitoring composting process. Despite the flaws, our work can be favorable to understand dynamic changes of bacterial and fungal community in composting process. Besides, some of the key species for nitrogen cycle or organic material degradation would be potential intervention manners for improving composting process.

5 Conclusion

Our results based on 16S and 18S rRNA gene sequencing illustrated the primary phylum in compost samples of during EFS included *Chloroflexi*, *Firmicutes* and *Proteobacteria* of bacteria, and *Ascomycota* of fungi. We also found decreased main pathogenic bacteria and increased fecal coliform. Bacterial and fungal diversity and composition significantly changed, while various physicochemical factors varied to fermentation maturity during the composting progress. Furthermore, we keystone

bacterial and fungal taxon were closely associated to physicochemical factors. Hence, management of physicochemical factors and microorganism could contribute to maturation rate and quality improvement during dairy manure ectopic fermentation.

Author Contributions: Conceptualization, P.G.; Formal analysis, P.G., X.H. and J.T.; Investiga-tion, L.W.; Software, L.W.; Resources, W.L.; Methodology, Y.Y. and E.J.; Writing - original draft, P.G. and Y.Y.; Writing—review & editing, Y.Y. and E.J.; Funding acquisition, E.J. All authors have read and agreed to the published version of the manuscript.

Funding: This research was funded by the Innovation Projects of Wuhan (CXJC201902).

Acknowledgments: We thank Institute of Animal Husbandry and Veterinary, Wuhan Academy of Agricultural Science and Shanghai Personal Biotechnology Co., Ltd. (China) for their assis-tance.

Conflicts of Interest: The authors declare no conflict of interest. The funders had no role in the design of the study; in the collection, analyses, or interpretation of data; in the writing of the manuscript, or in the decision to publish the results.

References

1. Qian XY, Shen GX, Wang ZQ, Chen XH, Zhao QJ, Bai YJ, et al. Application of dairy manure as fertilizer in dry land in East China: field monitoring and model estimation of heavy metal accumulation in surface soil. Environ Sci Pollut Res Int.

2020;27(29):36913-9. Epub 2020/06/25. doi: 10.1007/s11356-020-09786-x. PubMed PMID: 32577963.

2. Guan Y, Huang G, Liu L, Huang CZ, Zhai M. Ecological network analysis for an industrial solid waste metabolism system. Environ Pollut. 2019;244:279-87. Epub 2018/10/21. doi: 10.1016/j.envpol.2018.10.052. PubMed PMID: 30342368.

3. Shen Q, Sun H, Yao X, Wu Y, Wang X, Chen Y, et al. A comparative study of pig manure with different waste straws in an ectopic fermentation system with thermophilic bacteria during the aerobic process: Performance and microbial community dynamics. Bioresource technology. 2019;281:202-8. Epub 2019/03/02. doi: 10.1016/j.biortech.2019.01.029. PubMed PMID: 30822641.

4. Ye S, Shen Q, Li Y, Sun H, Yao X, Wu Y, et al. Analysis of the microbial community of pig manure fermented with straw litter in an ectopic fermentation system. Journal of Agro-Environment Science. 2020;39(3):638-47. PubMed PMID: CSCD:6727380.

5. Westerath HS, Laister S, Winckler C, Knierim U. Exploration as an indicator of good welfare in beef bulls: An attempt to develop a test for on-farm assessment. Applied Animal Behaviour Science. 2009;116(2-4):126-33. doi: 10.1016/j.applanim.2008.08.012. PubMed PMID: WOS:000262739200004.

6. Shen Q, Tang J, Wang X, Li Y, Yao X, Sun H, et al. Fate of antibiotic resistance genes and metal resistance genes during the thermophilic fermentation of solid and liquid swine manures in an ectopic fermentation system. Ecotoxicol Environ Saf. 2021;213:111981. Epub 2021/02/17. doi: 10.1016/j.ecoenv.2021.111981. PubMed

PMID: 33592372.

7. Manyi-Loh CE, Mamphweli SN, Meyer EL, Makaka G, Simon M, Okoh AI. An Overview of the Control of Bacterial Pathogens in Cattle Manure. International journal of environmental research and public health. 2016;13(9). Epub 2016/08/30. doi: 10.3390/ijerph13090843. PubMed PMID: 27571092; PubMed Central PMCID: PMCPMC5036676.

8. Pandey P, Chiu C, Miao M, Wang Y, Settles M, Del Rio NS, et al. 16S rRNA analysis of diversity of manure microbial community in dairy farm environment. PLoS One. 2018;13(1):e0190126. Epub 2018/01/06. doi: 10.1371/journal.pone.0190126. PubMed PMID: 29304047; PubMed Central PMCID: PMCPMC5755784.

9. Chen Q, Liu B, Zhu Y, Liu G, Che J, Wang J, et al. Bacterial community diversity of litters at different depths in microbial fermentation bed. Journal of Agro-Environment Science. 2019;38(10):2412-9. PubMed PMID: CSCD:6600113.

10. Guo H, Zhu C, Geng B, Liu X, Ye J, Tian Y, et al. Improved fermentation performance in an expanded ectopic fermentation system inoculated with thermophilic bacteria. Bioresource technology. 2015;198:867-75. Epub 2015/10/16. doi: 10.1016/j.biortech.2015.09.105. PubMed PMID: 26469215.

11. Yang X, Geng B, Zhu C, Li H, He B, Guo H. Fermentation performance optimization in an ectopic fermentation system. Bioresource technology. 2018;260:329-37. Epub 2018/04/11. doi: 10.1016/j.biortech.2018.03.101. PubMed PMID: 29635213.

12. Soepranianondo K, Wardhana DK, Budiarto, Diyantoro. Analysis of bacterial

contamination and antibiotic residue of beef meat from city slaughterhouses in East Java Province, Indonesia. Vet World. 2019;12(2):243-8. Epub 2019/05/02. doi: 10.14202/vetworld.2019.243-248. PubMed PMID: 31040565; PubMed Central PMCID: PMCPMC6460862.

13. Tok S, de Haan K, Tseng D, Usanmaz CF, Ceylan Koydemir H, Ozcan A. Early detection of E. coli and total coliform using an automated, colorimetric and fluorometric fiber optics-based device. Lab Chip. 2019;19(17):2925-35. Epub 2019/08/03. doi: 10.1039/c9lc00652d. PubMed PMID: 31372607.

14. Ministry of Agriculture and Rural Areas of People's Republic of China, Chinese National Agricultural Organic Fertilizer Standard (NY525-2012).

15. Magoč T, Salzberg SL. FLASH: fast length adjustment of short reads to improve genome assemblies. Bioinformatics. 2011;27(21):2957-63. Epub 2011/09/10. doi: 10.1093/bioinformatics/btr507. PubMed PMID: 21903629; PubMed Central PMCID: PMCPMC3198573.

16. He Y, Caporaso JG, Jiang XT, Sheng HF, Huse SM, Rideout JR, et al. Stability of operational taxonomic units: an important but neglected property for analyzing microbial diversity. Microbiome. 2015;3:20. Epub 2015/05/23. doi: 10.1186/s40168-015-0081-x. PubMed PMID: 25995836; PubMed Central PMCID: PMCPMC4438525.

17. Edgar RC. UPARSE: highly accurate OTU sequences from microbial amplicon reads. Nat Methods. 2013;10(10):996-8. Epub 2013/08/21. doi: 10.1038/nmeth.2604. PubMed PMID: 23955772.

18. Westcott SL, Schloss PD. OptiClust, an Improved Method for Assigning

Amplicon-Based Sequence Data to Operational Taxonomic Units. mSphere. 2017;2(2). Epub 2017/03/16. doi: 10.1128/mSphereDirect.00073-17. PubMed PMID: 28289728; PubMed Central PMCID: PMCPMC5343174.

19. Friedman J, Alm EJ. Inferring correlation networks from genomic survey data. PLoS computational biology. 2012;8(9):e1002687. Epub 2012/10/03. doi: 10.1371/journal.pcbi.1002687. PubMed PMID: 23028285; PubMed Central PMCID: PMCPMC3447976.

20. Francy DS, Myers DN, Metzker KD. Escherichia coli and fecal-coliform bacteria as indicators of recreational water quality. Report. 1993 93-4083.

21. Chen Q, Wang J, Zhang H, Shi H, Liu G, Che J, et al. Microbial community and function in nitrogen transformation of ectopic fermentation bed system for pig manure composting. Bioresource technology. 2021;319:124155. Epub 2020/10/10. doi: 10.1016/j.biortech.2020.124155. PubMed PMID: 33035862.

22. Liu T, Wang M, Awasthi MK, Chen H, Awasthi SK, Duan Y, et al. Measurement of cow manure compost toxicity and maturity based on weed seed germination. Journal of Cleaner Production. 2020;245. doi: 10.1016/j.jclepro.2019.118894. PubMed PMID: WOS:000503739400082.

23. Yang X, Song Z, Zhou S, Guo H, Geng B, Peng X, et al. Insights into functional microbial succession during nitrogen transformation in an ectopic fermentation system. Bioresource technology. 2019;284:266-75. Epub 2019/04/06. doi: 10.1016/j.biortech.2019.03.135. PubMed PMID: 30952054.

24. Wang Y, Sheng HF, He Y, Wu JY, Jiang YX, Tam NF, et al. Comparison of the

levels of bacterial diversity in freshwater, intertidal wetland, and marine sediments by using millions of illumina tags. Appl Environ Microbiol. 2012;78(23):8264-71. Epub 2012/09/25. doi: 10.1128/aem.01821-12. PubMed PMID: 23001654; PubMed Central PMCID: PMCPMC3497375.

25. Wang K, Wu Y, Li W, Wu C, Chen Z. Insight into effects of mature compost recycling on N(2)O emission and denitrification genes in sludge composting. Bioresource technology. 2018;251:320-6. Epub 2018/01/01. doi: 10.1016/j.biortech.2017.12.077. PubMed PMID: 29289876.

26. de Gannes V, Eudoxie G, Hickey WJ. Prokaryotic successions and diversity in composts as revealed by 454-pyrosequencing. Bioresource technology. 2013;133:573-80. Epub 2013/03/12. doi: 10.1016/j.biortech.2013.01.138. PubMed PMID: 23475177.

27. Huhe, Jiang C, Wu Y, Cheng Y. Bacterial and fungal communities and contribution of physicochemical factors during cattle farm waste composting. Microbiologyopen. 2017;6(6). Epub 2017/07/25. doi: 10.1002/mbo3.518. PubMed PMID: 28736905; PubMed Central PMCID: PMCPMC5727367.

28. Zhang L, Zhang H, Wang Z, Chen G, Wang L. Dynamic changes of the dominant functioning microbial community in the compost of a 90-m(3) aerobic solid state fermentor revealed by integrated meta-omics. Bioresource technology. 2016;203:1-10. doi: 10.1016/j.biortech.2015.12.040. PubMed PMID: WOS:000368016000001.

29. Zhang L, Ma H, Zhang H, Xun L, Chen G, Wang L. Thermomyces lanuginosus is the dominant fungus in maize straw composts. Bioresource technology. 2015;197:266-75. doi: 10.1016/j.biortech.2015.08.089. PubMed PMID: WOS:000361828000036.

30. Zhu N, Zhu Y, Kan Z, Li B, Cao Y, Jin H. Effects of two-stage microbial inoculation on organic carbon turnover and fungal community succession during co-composting of cattle manure and rice straw. Bioresource technology. 2021;341:125842. Epub 2021/09/02. doi: 10.1016/j.biortech.2021.125842. PubMed PMID: 34469819.

31. Tian X, Yang T, He J, Chu Q, Jia X, Huang J. Fungal community and cellulose-degrading genes in the composting process of Chinese medicinal herbal residues. Bioresource technology. 2017;241:374-83. Epub 2017/06/05. doi: 10.1016/j.biortech.2017.05.116. PubMed PMID: 28578278.

32. Rocha Vieira F, Andrew Pecchia J. Fungal community assembly during a high-temperature composting under different pasteurization regimes used to elaborate the Agaricus bisporus substrate. Fungal Biol. 2021;125(10):826-33. Epub 2021/09/20. doi: 10.1016/j.funbio.2021.05.004. PubMed PMID: 34537178.

33. Hu T, Wang X, Zhen L, Gu J, Zhang K, Wang Q, et al. Effects of inoculating with lignocellulose-degrading consortium on cellulose-degrading genes and fungal community during co-composting of spent mushroom substrate with swine manure. Bioresource technology. 2019;291:121876. Epub 2019/08/05. doi: 10.1016/j.biortech.2019.121876. PubMed PMID: 31377509.

34. Xu J, Jiang Z, Li M, Li Q. A compost-derived thermophilic microbial consortium enhances the humification process and alters the microbial diversity during composting. J Environ Manage. 2019;243:240-9. Epub 2019/05/18. doi: 10.1016/j.jenvman.2019.05.008. PubMed PMID: 31100660.

35. Wang K, Yin X, Mao H, Chu C, Tian Y. Changes in structure and function of fungal

community in cow manure composting. Bioresource technology. 2018;255:123-30. Epub 2018/02/08. doi: 10.1016/j.biortech.2018.01.064. PubMed PMID: 29414157.

36. Wang X, Kong Z, Wang Y, Wang M, Liu D, Shen Q. Insights into the functionality of fungal community during the large scale aerobic co-composting process of swine manure and rice straw. J Environ Manage. 2020;270:110958. Epub 2020/07/30. doi: 10.1016/j.jenvman.2020.110958. PubMed PMID: 32721362.

37. Basotra N, Kaur B, Di Falco M, Tsang A, Chadha BS. Mycothermus thermophilus (Syn. Scytalidium thermophilum): Repertoire of a diverse array of efficient cellulases and hemicellulases in the secretome revealed. Bioresource technology. 2016;222:413-21. doi: 10.1016/j.biortech.2016.10.018. PubMed PMID: WOS:000386243600050.

38. Jiao S, Chen W, Wei G. Biogeography and ecological diversity patterns of rare and abundant bacteria in oil-contaminated soils. Mol Ecol. 2017;26(19):5305-17. Epub 2017/07/01. doi: 10.1111/mec.14218. PubMed PMID: 28665016.

39. Banerjee S, Walder F, Büchi L, Meyer M, Held AY, Gattinger A, et al. Agricultural intensification reduces microbial network complexity and the abundance of keystone taxa in roots. The ISME journal. 2019;13(7):1722-36. Epub 2019/03/10. doi: 10.1038/s41396-019-0383-2. PubMed PMID: 30850707; PubMed Central PMCID: PMCPMC6591126.

40. Li P, Liu M, Ma X, Wu M, Jiang C, Liu K, et al. Responses of microbial communities to a gradient of pig manure amendment in red paddy soils. Sci Total Environ. 2020;705:135884. Epub 2019/12/11. doi: 10.1016/j.scitotenv.2019.135884. PubMed PMID: 31818573.

41. Feng K, Zhang Z, Cai W, Liu W, Xu M, Yin H, et al. Biodiversity and species competition regulate the resilience of microbial biofilm community. Mol Ecol. 2017;26(21):6170-82. Epub 2017/09/20. doi: 10.1111/mec.14356. PubMed PMID: 28926148.

Figure 1

Click here to access/download;Figure;Figure 1.tif

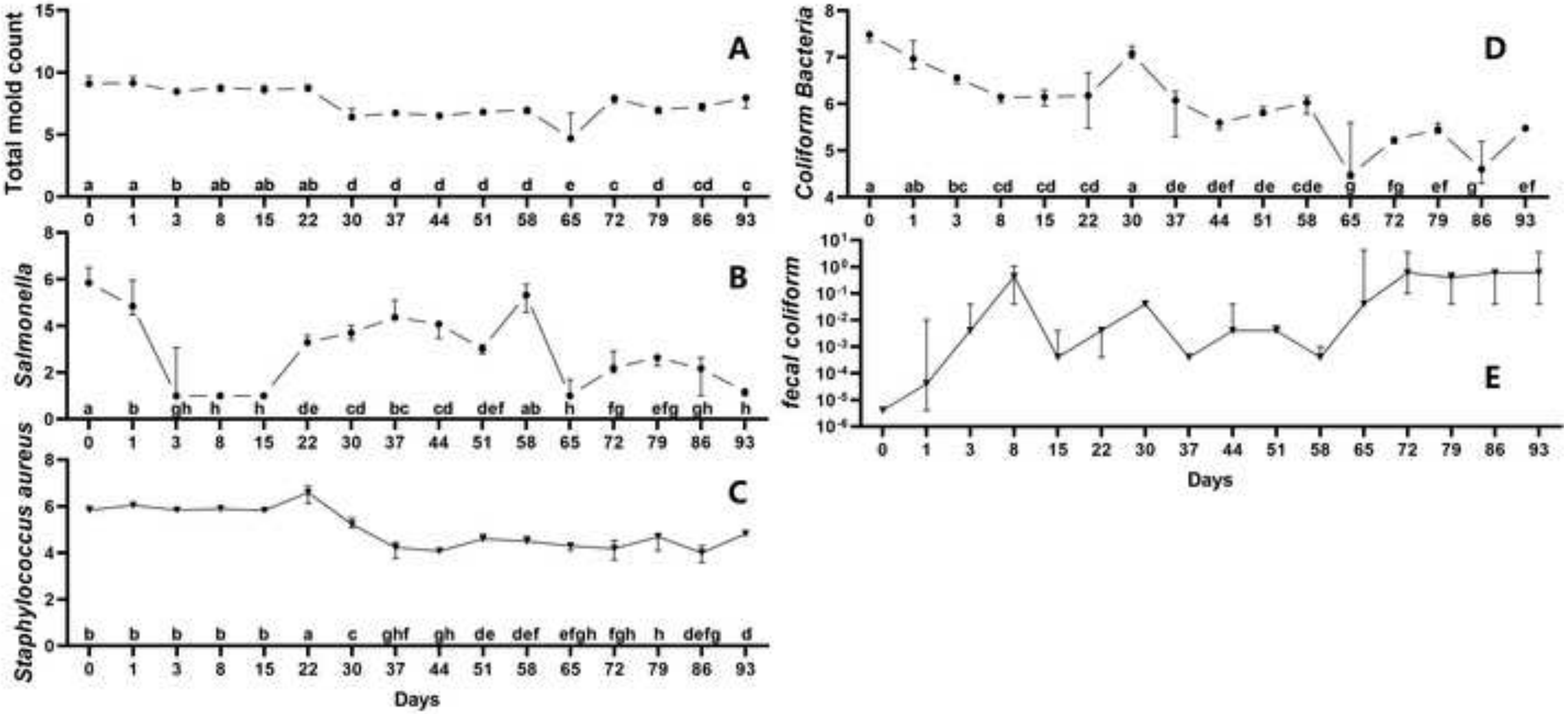

Figure 2

Click here to access/download;Figure;Figure 2.tif

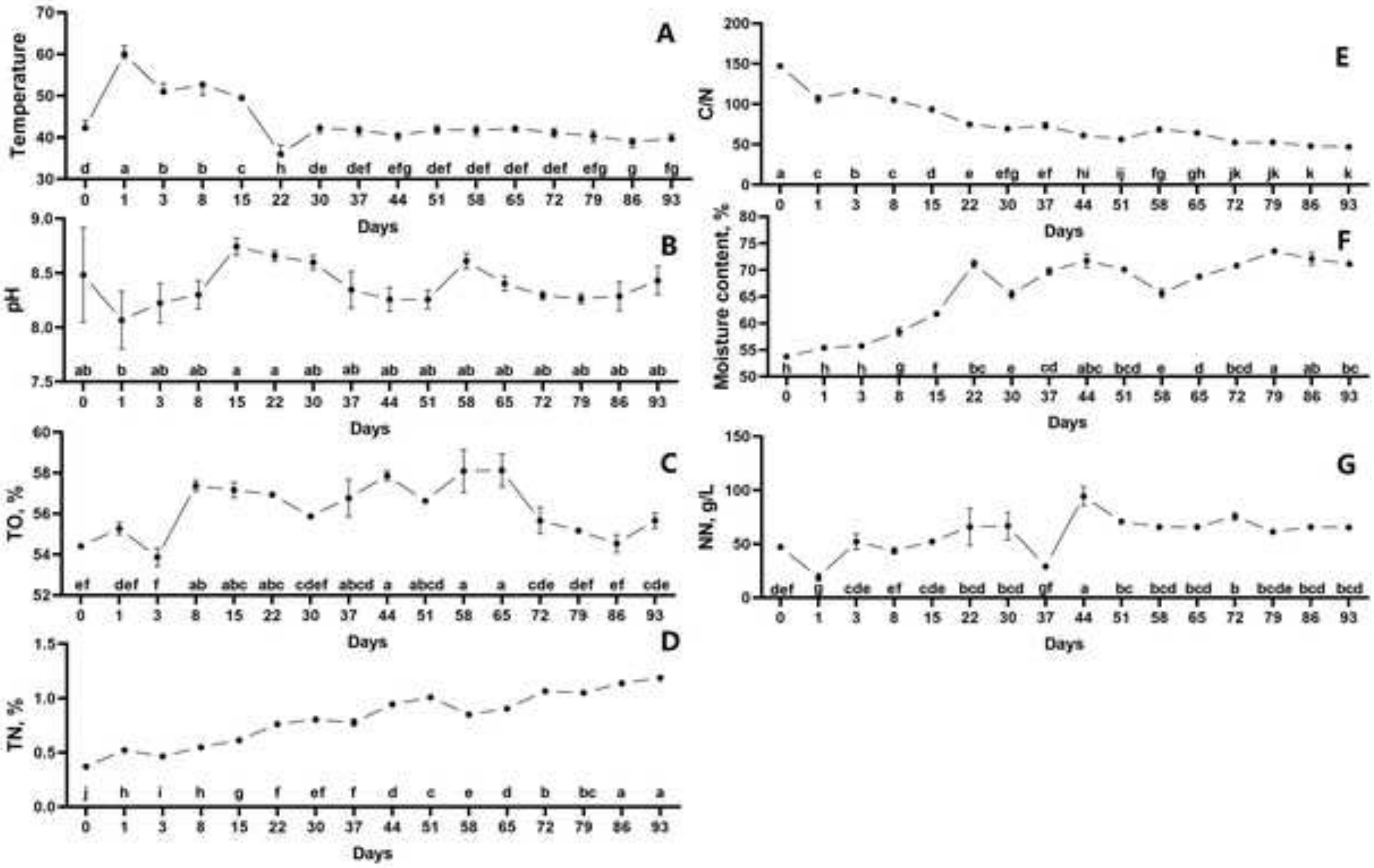

Figure 3

Click here to access/download;Figure;Figure 3.tif

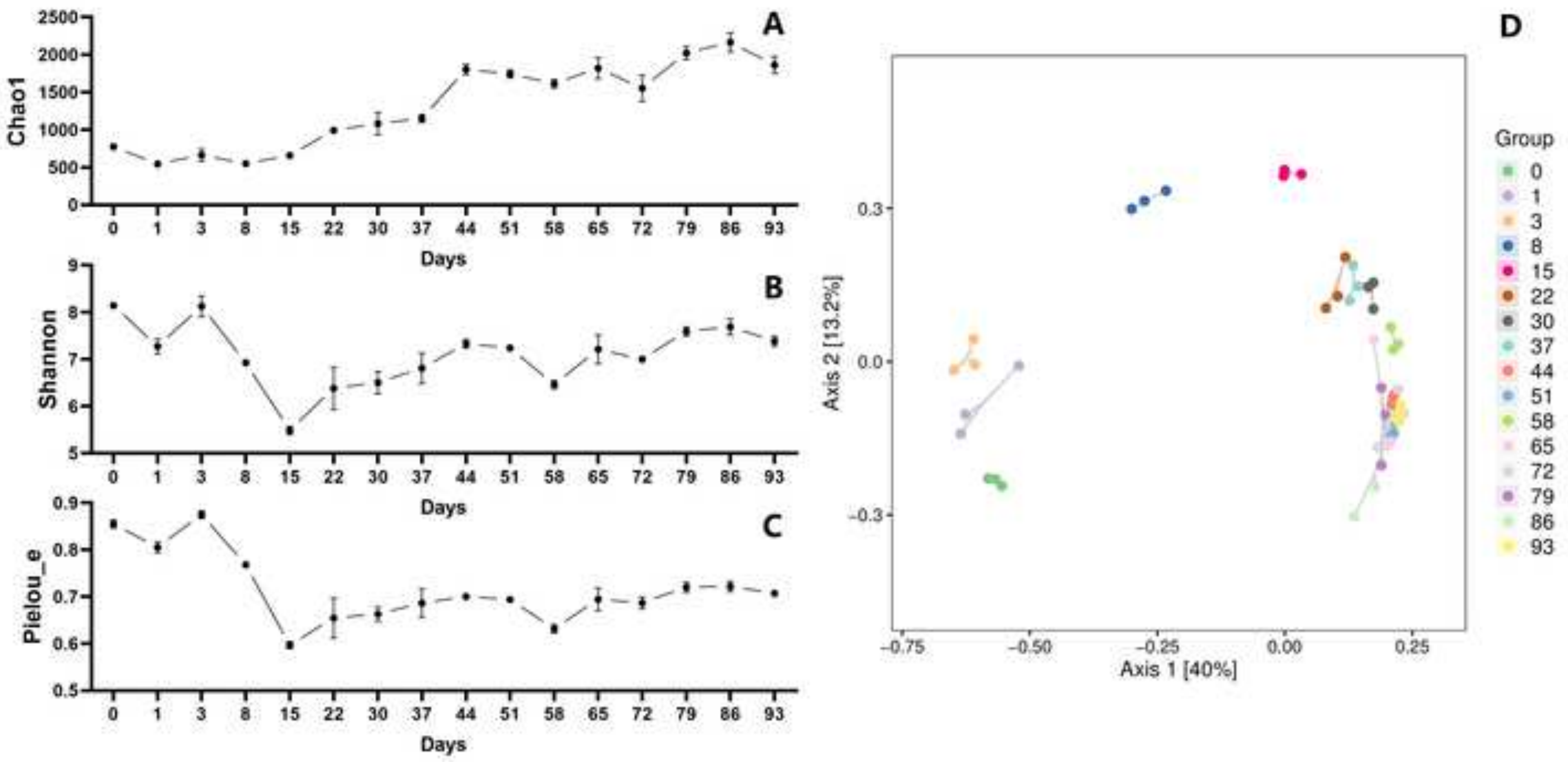

Figure 4

Click here to access/download;Figure;Figure 4.tif

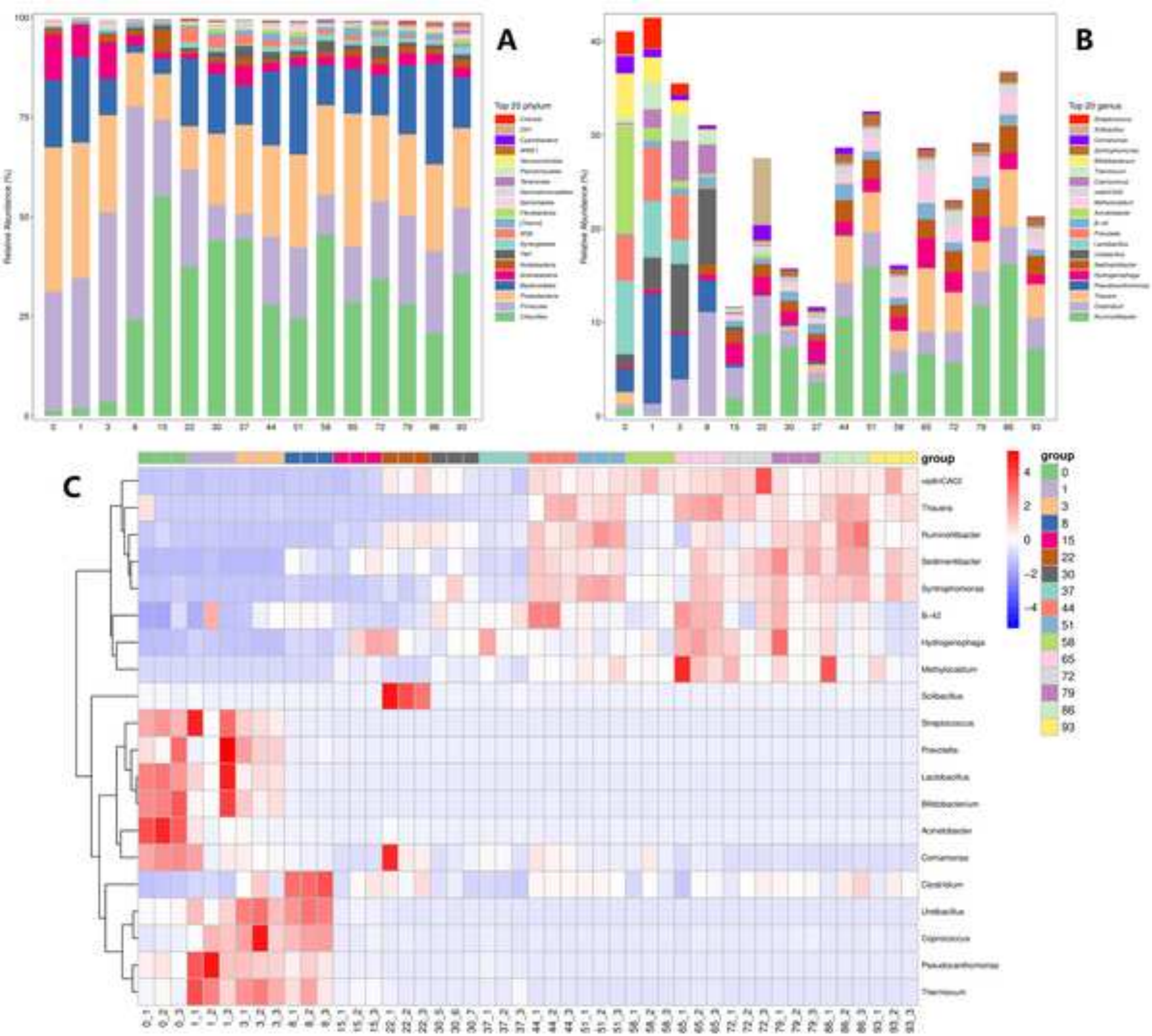

Figure 5

Click here to access/download;Figure;Figure 5.tif

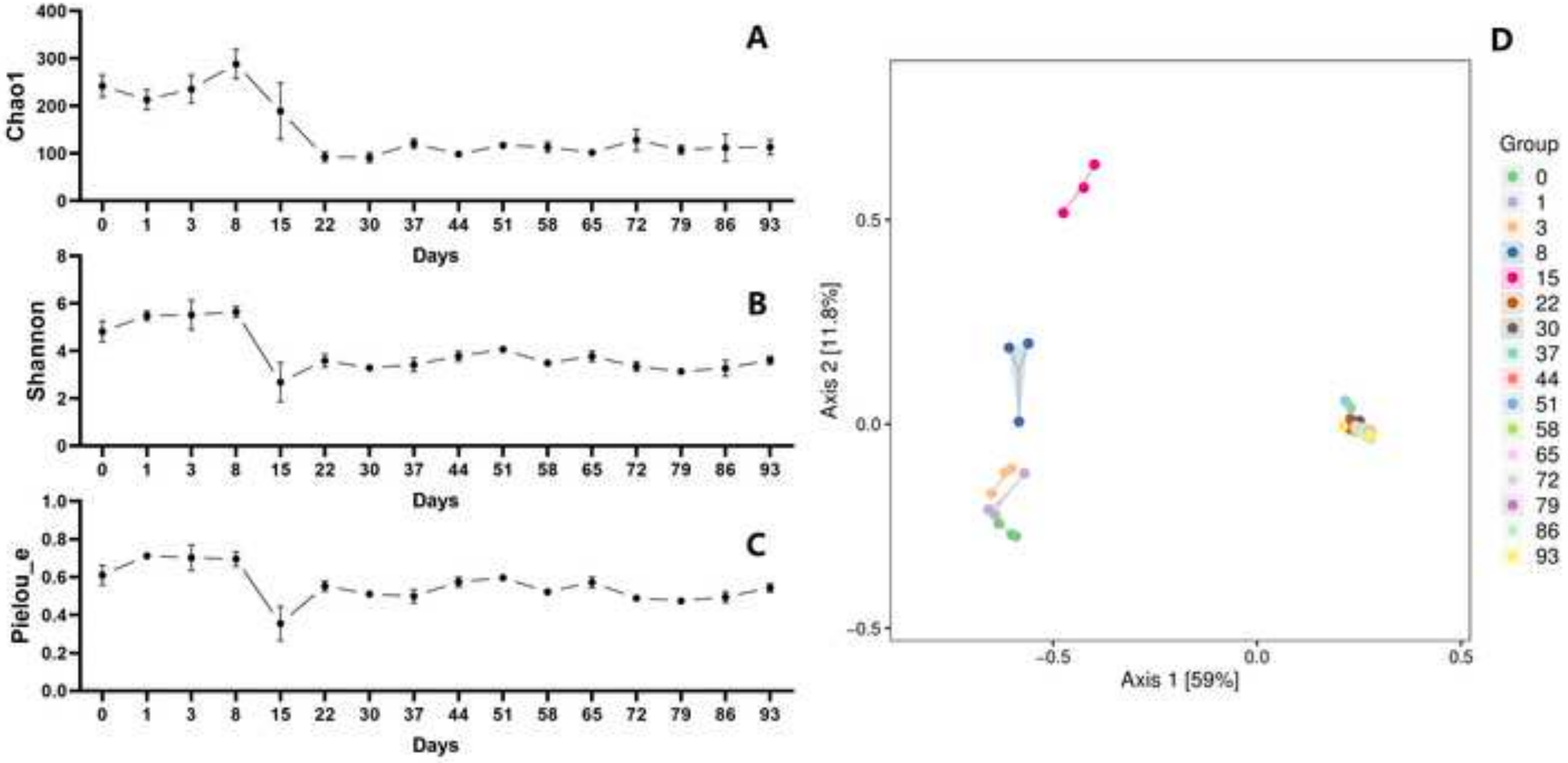

Figure 6

Click here to access/download;Figure;Figure 6.tif

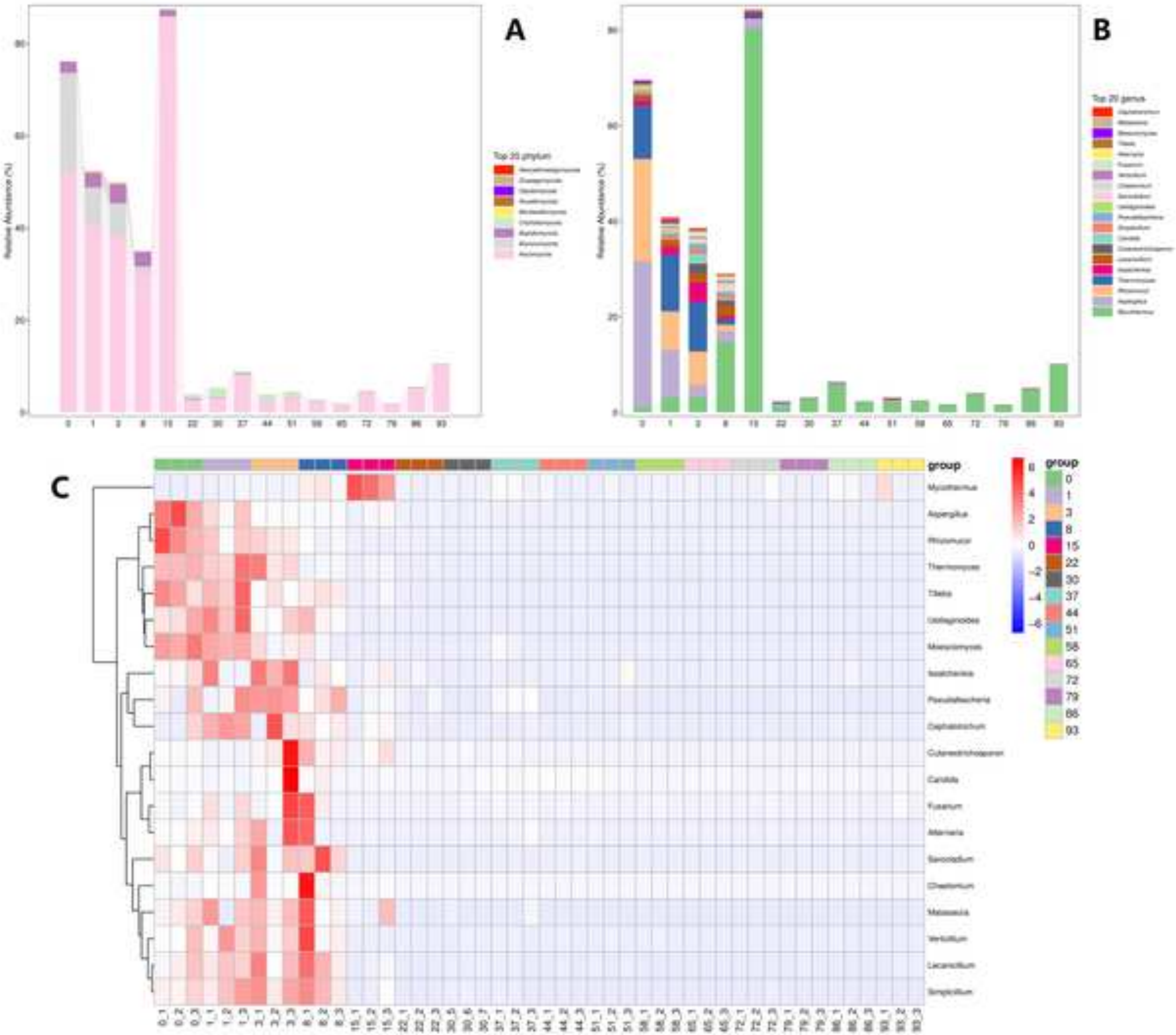

Figure 7 Click here to access/download;Figure;Figure 7.tif

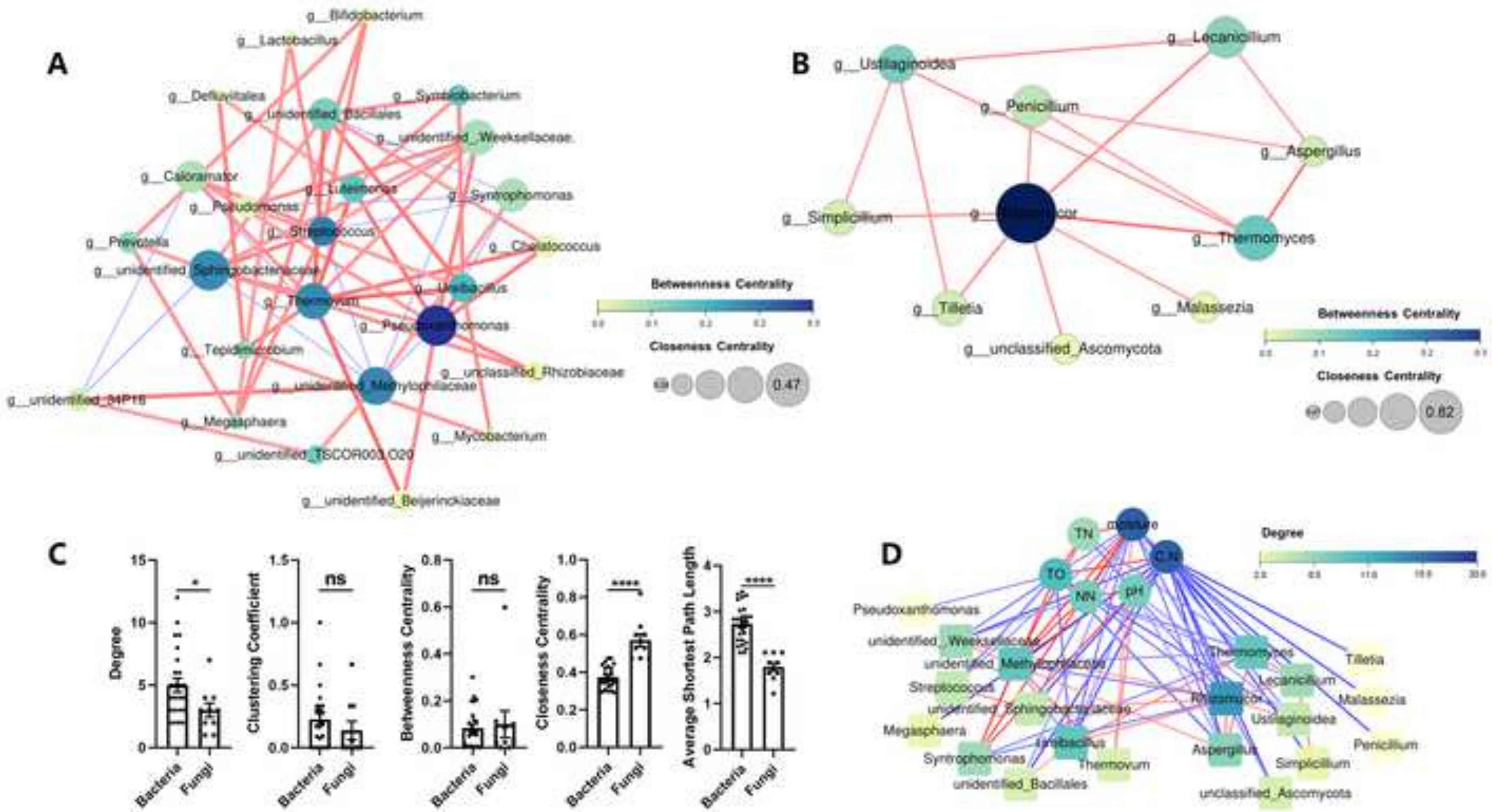

Figure 8

Click here to access/download;Figure;Figure 8.tif

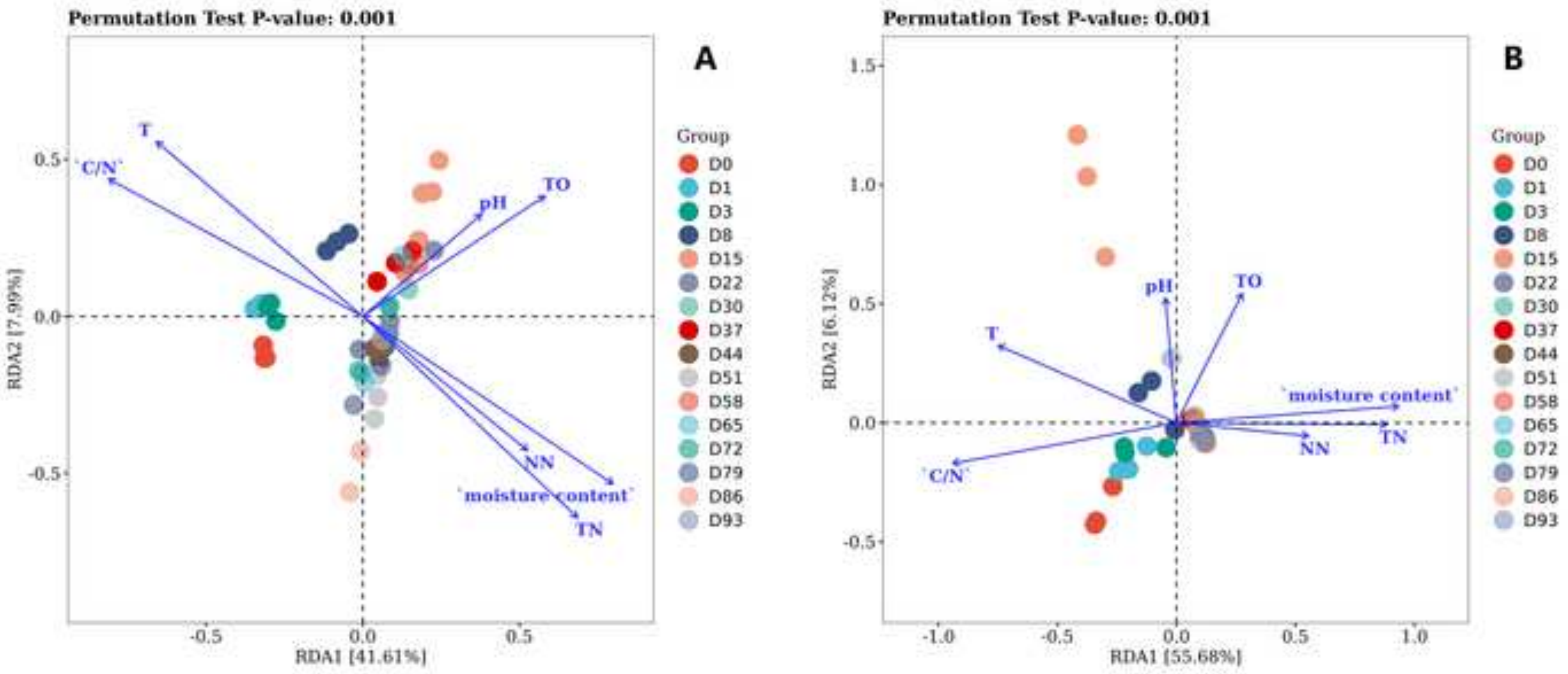

Supplement: Attachment — Submitted filename: PONE-D-22-13572_reviewer.pdf [file pone.0276920.s002.pdf]
