# Supplementary material for: Changes of bacterial and fungal communities and relationship between keystone taxon and physicochemical factors during dairy manure ectopic fermentation
Source: PLoS One. 2022 Dec 19;17(12):e0276920. doi: 10.1371/journal.pone.0276920 (PMC9762577; doi:10.1371/journal.pone.0276920)
Supplement: Attachment — Submitted filename: Response to Reviewers 1001.docx [file pone.0276920.s004.docx]

**Reviewer #1**: Dear Authors,

The manuscript has been well revised with no further comments while reading it. Now it can be accepted for possible publication.

Response: Thanks for your constructive advice and instruction during the peer review work, which is crucial and vital for our improving the manuscripts quality. We are also very appreciated for your affirmation and acceptance comments.

**Reviewer #2**: line 26: rise instead of rose, please correct it. please check all typographical errors.

Response: Thanks for the comments and we are sorry for the writing mistake. We have revised it, please see line 26.

We have re-read our work and carefully check the manuscripts, and we have revised same typographical errors. Please see Line 23, Line 61, Line 107, Line 110, Line 338, Line 343, Line 316, Line 362, Line 364, Line 405 and Line 442.

please improve the quality of the figures, they are invisible.

Response: Thanks for your constructive advice and we are sorry for mistake and negligence during plotting. We have re-plot invisible figures, please see figure files.
